# Arteriovenous malformation of the pancreas: a case report

**DOI:** 10.1186/s40792-016-0133-x

**Published:** 2016-02-01

**Authors:** Tsuyoshi Abe, Nobuyasu Suzuki, Junichirou Haga, Ayaka Azami, Yukitoshi Todate, Mitsuru Waragai, Atai Sato, Yoshinao Takano, Kenji Kawakura, Shigeki Imai, Hideo Sakuma, Yasushi Teranishi

**Affiliations:** Department of Surgery, Southern Tohoku Research Institute for Neuroscience, 7-115 Yatsuyamada, Koriyama, 963-8563 Japan; Department of Radiology, Southern Tohoku Research Institute for Neuroscience, 7-115 Yatsuyamada, Koriyama, 963-8563 Japan; Department of Pathology, Southern Tohoku Research Institute for Neuroscience, 7-115 Yatsuyamada, Koriyama, 963-8563 Japan

**Keywords:** Pancreatic arteriovenous malformation, Pancreaticoduodenectomy, 3D image

## Abstract

Arteriovenous malformation (AVM) of the pancreas is uncommon in the gastrointestinal tract. We present a case of AVM of the pancreatic head in a 59-year-old male. He was admitted to a hospital with hematemesis and tarry stool and referred to our hospital in March 2014 on the diagnosis of pancreatic artery pseudoaneurysm. A computed tomography scan showed the presence of irregular dilated and/or stenotic vessels with meandering in the pancreatic head. Magnetic resonance imaging showed strong enhancement of the conglomeration in the pancreatic head. Selective angiography showed the proliferation of a vascular network in the pancreatic head and an early visualization of the portal vein at the arterial phase. The patient qualified for surgery with a preoperative diagnosis of AVM of the pancreatic head. We performed pylorus-preserving pancreaticoduodenectomy. The histological results confirmed the presence of irregular dilated tortuous arteries and veins in the pancreatic head. Surgical treatment may represent definitive management of symptomatic AVM.

## Background

An arteriovenous malformation (AVM) is a complex tangle of abnormal arteries and veins linked by one or more direct connections called a fistula or shunt. The number of reported cases of AVM in digestive organs has increased in recent years because of the widespread use of imaging modalities such as angiography, computed tomography (CT), magnetic resonance imaging, and color Doppler ultrasonography. However, the incidence of pancreatic AVM remains extremely low, with fewer than 200 cases reported in the literature since Halpern described the first case in 1968 [1]. Majority of the patients with pancreatic AVM remain asymptomatic, but some patients present with abdominal pain or gastrointestinal bleeding.

A definitive diagnosis is established using angiographic evaluation. Surgery is regarded as the definitive treatment for pancreatic AVM. Here, we summarize the clinical course and radiological findings of a patient with pancreatic AVM and include a review of the associated literature.

## Case presentation

A 59-year-old male was admitted to another hospital in March 2014 with symptoms of hematemesis and tarry stool. He had a history of hypertension, cardiac infarction, and aortic regurgitation. Laboratory data collected on admission revealed anemia (hemoglobin concentration, 6.4 g/dl), and he received a transfusion of four units of packed red blood cells. A CT scan showed the presence of irregular dilated arteries in the pancreatic head, and he was diagnosed with pancreatic artery pseudoaneurysm presenting with multiple arterial feeder vessels.

**Fig. 1:**
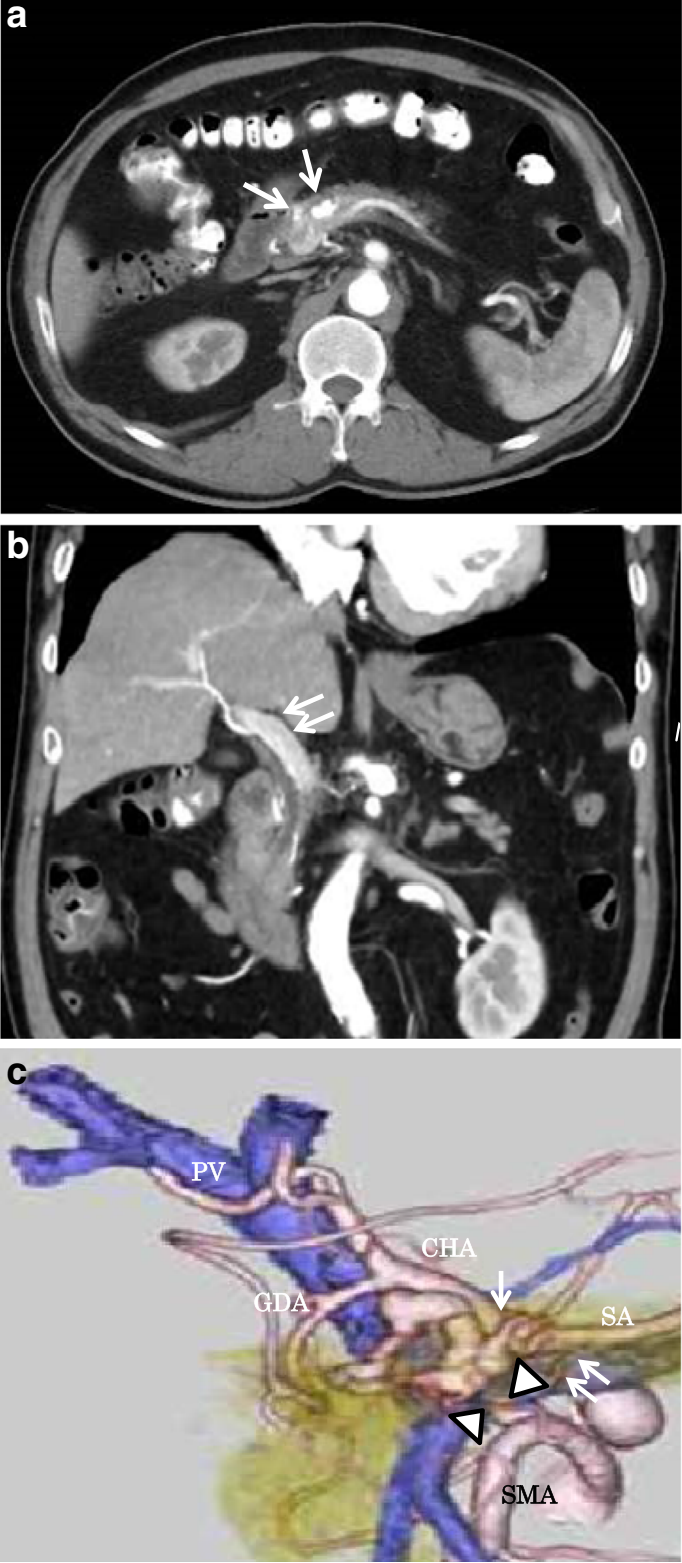
Computed tomography scan showed irregular dilatation with meandering in the pancreatic head (*single arrow*; **a**) and the early appearance of the portal vein at the arterial phase (*double arrows*; **b**). Three-dimensional imaging showed feeders from the superior mesenteric artery (SMA) (*arrow heads*), celiac artery (*single arrow*), and splenic artery (*double arrows*) (**c**). *PV* portal vein, *CHA* common hepatic artery, *GDA* gastroduodenal artery, *SA* splenic artery

**Fig. 2:**
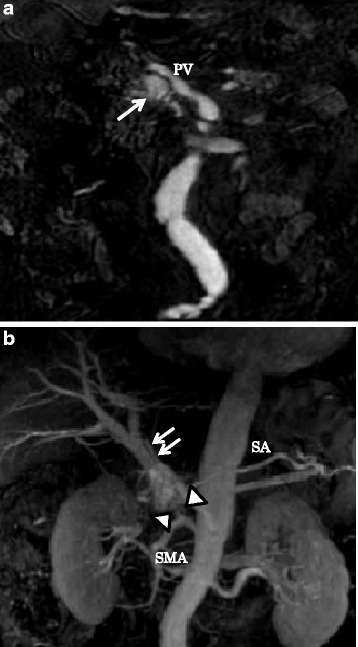
Magnetic resonance imaging showed strong enhancement of the conglomeration in the pancreatic head (*single arrow*; **a**). Reformed maximum intensity projection imaging showed feeders from the superior mesenteric artery (SMA) (*arrow heads*) with the early appearance of the portal vein (*double arrows*) in the arterial phase (**b**). *PV* portal vein, *SA* splenic artery

**Fig. 3:**
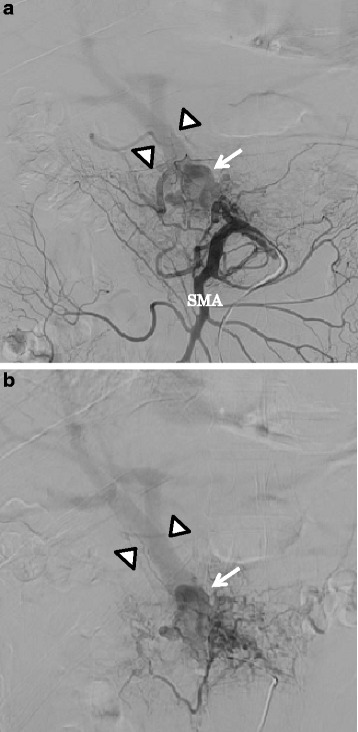
Superior mesenteric (**a**) and selective superior mesenteric angiography (**b**) showing dilated arteries and a racemose vascular network in the pancreas head (*single arrow*). Early visualization of the portal vein (*arrow heads*) was also demonstrated in this phase. *SMA* superior mesenteric artery

The patient was referred to our hospital for treatment. On admission, a CT scan showed the presence of meandering irregular dilated vessels in the pancreatic head (Fig. 1a) and an early appearance of the portal vein at the arterial phase (Fig. 1b). Further evaluation using 3D imaging showed feeder vessels from the superior mesenteric, celiac, and splenic arteries (Fig. 1c). Magnetic resonance imaging showed the strong enhancement of the conglomeration in the pancreatic head (Fig. 2a), and reformed maximum intensity projection imaging showed feeder vessels from the superior mesenteric and celiac arteries with an early appearance of the portal vein at the arterial phase (Fig. 2b). Selective angiography showed the proliferation of a vascular network in the pancreatic head and early visualization of the portal vein at the arterial phase (Fig. 3a, b). Color Doppler imaging revealed a mosaic-like structure comprising pulsatile waves. The preoperative diagnosis was AVM of the pancreatic head.

**Fig. 4:**
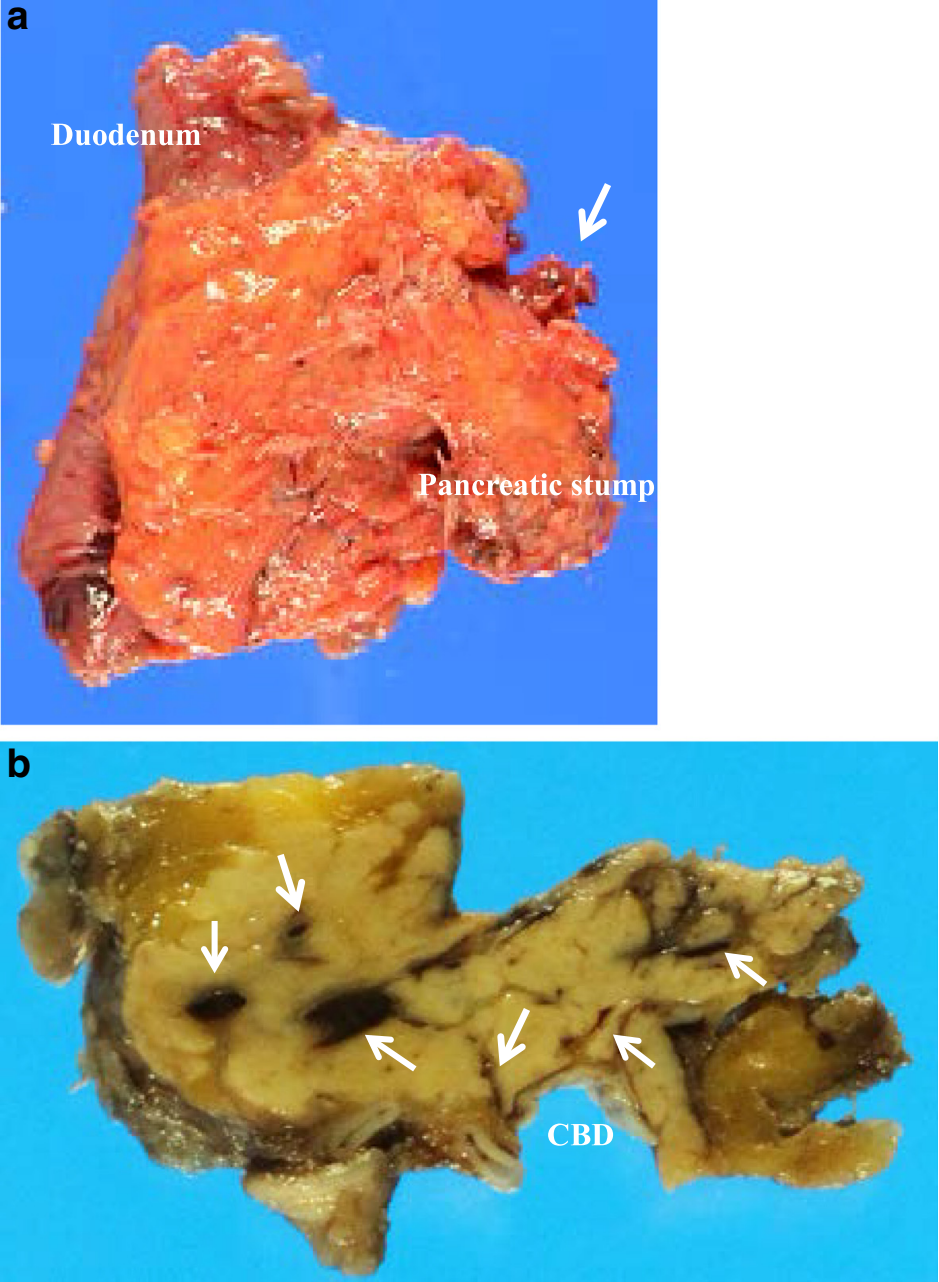
The resected specimen showed that the pancreatic head was normal in appearance, except the presence of dilated drainage veins (*single arrow*) at the top of the pancreatic head (**a**). Serial sections of the resected specimens revealed dilated and tortuous vessels (*single arrow*) in the pancreatic head (**b**). *CBD* common bile duct

**Fig. 5:**
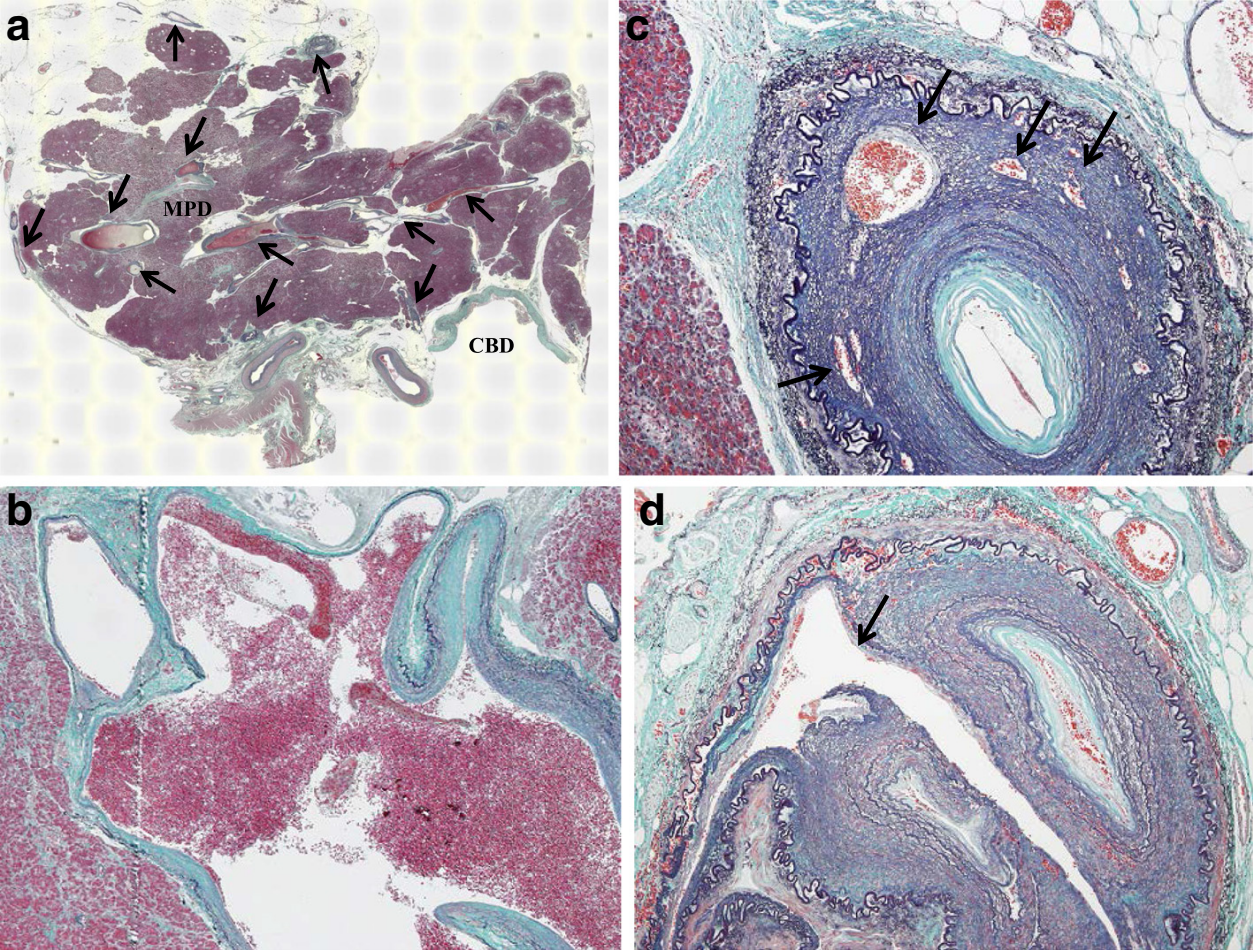
Histological examination revealed dilated and tortuous vessels (*single arrow*) in the pancreatic subcapsular tissue and parenchyma and surrounding fat tissue (**a**); these vessels in the lesion that cannot be morphologically differentiated from either arteries or veins (**b**), numerous arteries have thickened walls, accompanied with severed elastic fibers and various dilated capillaries in the vessel media (*single arrow*) (**c**), and dissections were found in outer media of the arteries (*single arrow*) (**d**). *CBD* common bile duct, *MPD* main pancreatic duct

The intraoperative examination revealed that there were no abnormal vessels and/or collateral vessels on the surface or around the pancreas except dilated drainage veins at the top of the pancreatic head (Fig. 4a). But, serial sections of the resected specimens revealed dilated and tortuous vessels in the pancreatic head (Fig 4b). Four feeder arteries from the superior mesenteric artery and two feeder arteries from the common hepatic artery were detected during the pancreatectomy. We performed pylorus-preserving pancreaticoduodenectomy. Duration of the operation was 11 h 59 min and amount of blood loss was 1950 ml. Histological examination revealed the conglomeration of irregular dilated and tortuous arteries and veins in the pancreatic subcapsular tissue and parenchyma and surrounding fat tissue (Fig. 5a, b); numerous arteries have thickened walls, accompanied with severed elastic fibers and various dilated capillaries in the vessel media (Fig. 5c), with frequently observed arterial dissection (Fig. 5d). The patient experienced a postoperative pancreatic fistula (An International Study Group of Postoperative Pancreatic Fistula grade B), but he was discharged on postoperative day 73. He remains healthy and disease-free 19 months after surgery.

### Discussion

Various hypotheses have been proposed to explain the origin of AVM. Lande et al. [2] speculated that a loss of regulation of the sphincter mechanism at the arteriolar–capillary junction results in an overflow of the arterial blood into the capillaries and venules, and eventually, in the formation of an arteriovenous shunt. Cases of pancreatic AVM are either congenital or acquired. Congenital AVMs are caused by the abnormal development of the arteriovenous plexus in the embryo, whereas acquired AVMs are caused by pancreatitis, tumors, or trauma. Among the reported cases, 90.5 % of the cases were considered to be of congenital origin, including patients with Osler–Weber–Rendu disease (OWRD) [3]. Most cases of pancreatic AVM outside of Japan are associated with OWRD and are known to be related to the visceral angiodysplasia of hereditary hemorrhagic telangiectasia. Pancreatic AVM in Japan may be different than that in other countries. However, the present case appeared to be of congenital origin because of the lack of pancreatitis, or a history of tumor or trauma. Our patient used an anti-platelet agent (Bayaspirin®). We cannot deny the possibility of bleeding from the gastrointestinal mucosa being caused by the use of an anti-platelet agent, but we considered that there was no relationship between the anti-platelet agent and pancreatic AVM.

In an AVM case series reported by Song et al. [4], the median age at diagnosis was 50 years (range, 7 months to 75 years) with an Asian (78.3 %) and male (88.4 %) predominance. The most common symptoms were gastrointestinal bleeding (49.3 %) and abdominal pain with or without back pain (40.6 %), although 18.8 % were asymptomatic. The portion of the pancreas with most frequent involvement is the head (59.4 %), followed by the body and tail (33.3 %) and the entire pancreas (7.2 %).

Mechanisms of gastrointestinal bleeding can be classified into four types: (1) bleeding from a duodenal ulcer due to ischemic injury of the duodenal mucosa by local infarction, resulting in abnormal vessels of pancreatic AVM; (2) bleeding from the pancreatic duct and/or the bile duct through the orifice of the ampulla of Vater; (3) bleeding from eroded vessels in the wall of the gastrointestinal tract by pancreatic AVM; and (4) bleeding from gastroesophageal varices because of portal hypertension due to pancreatic AVM. In our case, no ulcers or varices of the esophagus or stomach were found during the preoperative endoscopy, and no abnormalities were found in the intestine during the operation. Furthermore, no abnormalities were found in the bile duct of the resected specimen. Macroscopically, the exact point of bleeding could not be identified. But, histological examination revealed that dissections were found in the outer media of the arteries in the pancreatic head (Fig. 5d). Therefore, we assumed that the gastrointestinal bleeding in our patient may have originated from the pancreatic duct through the orifice of the ampulla of Vater.

Diagnosis is usually confirmed by imaging because the symptoms are nonspecific and the incidence of this entity is very low. Angiography is useful for the diagnosis of pancreatic AVM, with the findings of pancreatic AVM characterized by dilated and tortuous feeding arteries, a racemose intrapancreatic vascular network followed by a transient dense pancreatic stain, early venous filling into the portal vein, and early disappearance of the pancreatic stain [5]. Recent technological advances and the availability of many diagnostic imaging modalities such as CT, magnetic resonance imaging, and color Doppler ultrasonography have made the diagnosis of pancreatic AVM safer and more accurate. CT 3D imaging and reformed maximum intensity projection imaging have proven very useful for detecting feeders and planning operative procedures. Doppler ultrasonography can demonstrate mosaic lesions composed of pulsatile waves, although its wave is normally flat [6].

In an AVM case series reported by Song et al. [4], pancreatic resection was performed in 46.4 % patients, extended devascularization with or without splenectomy in 10.1 %, transarterial embolization (TAE) alone in 7.2 %, irradiation in 2.9 %, transjugular intrahepatic portosystemic shunt (TIPS) in 2.9 %, and no treatment in 21.7 %. Generally, pancreatic AVMs contain multiple feeder arteries, making it very difficult to achieve complete embolization or ligation. Although some authors have suggested performing preoperative TAE to reduce surgical risks by decreasing portal flow, the risk of recurrent bleeding always exists. Cases successfully treated with TAE alone were more common among patients without hemorrhage than in patients with hemorrhage. All patients with hemorrhage treated with TAE required surgery. Recurrent bleeding after TAE has been reported in up to 18–37 % of patients waiting for surgery [7–9]. It is important to be aware of complication risks caused by embolization such as bowel ischemia resulting from distal organ embolization [10]. Based on these findings, TAE may be indicated for selective cases such as patients who have a single feeder artery, who are at a high surgical risk, and/or who have no hemorrhage. The treatment of asymptomatic pancreatic AVM is still controversial. Some authors have recommended early surgical resection because the condition can progress to portal hypertension and lead to gastrointestinal bleeding or rupture of esophageal varices [11–13]. However, the natural course of asymptomatic pancreatic AVM has not yet been elucidated. The definitive treatment for symptomatic AVM of the pancreas is surgical resection because it eliminates the cause of pain and prevents the development of portal hypertension. Portal hypertension appears to be the major prognostic factor for pancreatic AVM because of its association with gastrointestinal bleeding and it sometimes not regressing after the resection of the pancreatic AVM [14]. When a patient is at a high surgical risk, TIPS and radiation therapy are alternative options [4]. There is only one report on long-term follow-up after a pancreatic resection for 11 patients with pancreatic AVMs [4]. The authors followed up with the patients with pancreatic AVM routinely through physical examinations, laboratory tests, and CTs. During a median follow-up time of 37 months, no patient had any major postoperative complications. They also analyzed 23 patients with pancreatic AVMs who had undergone pancreatic resections; of these, only one patient had died 10 months after pancreatic resection because of portal hypertension with repeated rupturing of the esophageal varices [4, 11]. The main cause of death was gastrointestinal hemorrhage associated with portal hypertension. In addition, we should take into account the relapse of AVM in the remnant pancreas after surgery. Therefore, the patients with pancreatic AVMs, either with or without treatment, should be routinely followed up using physical examinations, laboratory tests, CTs, and endoscopies to identify any varices associated with portal hypertension.

The patient presented here underwent pylorus-preserving pancreaticoduodenectomy and had a favorable outcome.

## Conclusions

In patients with symptomatic pancreatic AVM, surgical resection of the affected pancreas may be the only definitive treatment. Patients with portal hypertension are generally followed carefully because of the potential for the recurrence of gastrointestinal bleeding or rupture of esophageal varices.

## Consent

Written informed consent was obtained from the patient for the publication of this case report and any accompanying images. A copy of the written consent form is available for review of the Editor-in-Chief of this journal.
